# Prebiotic Inulin Supplementation and Peripheral Insulin Sensitivity in adults at Elevated Risk for Type 2 Diabetes: A Pilot Randomized Controlled Trial

**DOI:** 10.3390/nu13093235

**Published:** 2021-09-17

**Authors:** Cassie M. Mitchell, Brenda M. Davy, Monica A. Ponder, Ryan P. McMillan, Michael D. Hughes, Matthew W. Hulver, Andrew P. Neilson, Kevin P. Davy

**Affiliations:** 1 Department of Human Nutrition, Foods, and Exercise, Virginia Tech, Blacksburg, VA 24061, USA; casmitch@vt.edu (C.M.M.); bdavy@vt.edu (B.M.D.); mcmillr@vt.edu (R.P.M.); hulvermw@vt.edu (M.W.H.); 2 Translational Obesity Research Interdisciplinary Graduate Education Program, Virginia Tech, Blacksburg, VA 24061, USA; mponder@vt.edu; 3 Obesity and Diabetes Clinical Research Section, Phoenix Epidemiology and Clinical Research Branch, National Institute of Diabetes and Digestive and Kidney Diseases, Phoenix, AZ 85016, USA; 4 Department of Food Science and Technology, Virginia Tech, Blacksburg, VA 24060, USA; michah3@vt.edu; 5 Plants for Human Health Institute, North Carolina Research Campus, North Carolina State University, Kannapolis, NC 28081, USA; aneilso@ncsu.edu

**Keywords:** dietary fiber, prebiotics, inulin, diabetes, metabolism, gut microbiota

## Abstract

Prediabetes affects 84.1 million adults, and many will progress to type 2 diabetes (T2D). The objective of this proof-of-concept trial was to determine the efficacy of inulin supplementation to improve glucose metabolism and reduce T2D risk. Adults (*n* = 24; BMI: 31.3 ± 2.9 kg/m^2^; age: 54.4 ± 8.3 years) at risk for T2D were enrolled in this controlled feeding trial and consumed either inulin (10 g/day) or placebo (maltodextrin, 10 g/day) for six weeks. Assessments included peripheral insulin sensitivity, fasting glucose, and insulin, HOMA-IR, in vivo skeletal muscle substrate preference, *Bifidobacteria* copy number, intestinal permeability, and endotoxin concentrations. Participant retention was 92%. There were no baseline group differences except for fasting insulin (*p* = 0.003). The magnitude of reduction in fasting insulin concentrations with inulin (*p* = 0.003, inulin = Δ-2.9, placebo = Δ2.3) was attenuated after adjustment for baseline concentrations (*p =* 0.04). After adjusting for baseline values, reduction in HOMA-IR with inulin (inulin = Δ-0.40, placebo=Δ0.27; *p =* 0.004) remained significant. *Bifidobacteria* 16s increased (*p =* 0.04; inulin = Δ3.1e^9^, placebo = Δ-8.9e^8^) with inulin supplementation. Despite increases in gut *Bifidobacteria,* inulin supplementation did not improve peripheral insulin sensitivity. These findings question the need for larger investigations of inulin and insulin sensitivity in this population.

## 1. Introduction

The prevalence of type 2 diabetes (T2D) among U.S. adults is estimated to be 13%, and it is the 7th leading cause of deaths in the US [1]. Prediabetes is a condition defined by impaired glucose tolerance (IGT), impaired fasting glucose (IFG), or a hemoglobin A1c (HbA1c) above normal but below the threshold indicative of T2D [1,2]. In 2018, the estimated number of U.S. adults with prediabetes was 88 million, and of these individuals, only 15.3% reported that a healthcare provider informed them of this diagnosis [1]. Although individual awareness of prediabetes has more than doubled from 6.5% since 2005, most people remain unaware of their metabolic condition [1,3,4,5]. The rising prevalence of prediabetes and T2D is a national health problem that warrants research focused upon T2D prevention strategies [1,3,4]. 

Lifestyle modification strategies for prevention of T2D include weight loss of 5–10% total body weight, 30 min of moderate physical activity (PA) at least 5 days/week, and consumption of dietary patterns focused on fruits, vegetables, and whole grains [6,7]. However, the impact of specific dietary modifications and their subsequent influence on T2D risk are not well understood. Human and animal studies suggest that consumption of a western diet, characterized by a high intake of dietary fat and sugar, may negatively impact gut microbiome composition by reducing bacterial taxa associated with gut health, increase intestinal permeability, and lead to metabolic endotoxemia [8,9,10]. In turn, metabolic endotoxemia is associated with the development of a low-grade chronic inflammatory state, obesity, and insulin resistance in rodents [8,10,11]. In humans, fasting endotoxin concentrations are higher in individuals with prediabetes and T2D compared with normoglycemic individuals [12], and endotoxemia is associated with an increased risk of prevalent and incident T2D [13]. Importantly, endotoxin induces an inflammatory response, which reduces insulin signaling and glucose transport in human skeletal muscle [14]. Furthermore, low dose endotoxin, at concentrations consistent with metabolic endotoxemia, activate skeletal muscle toll-like receptor 4 and causes a state of metabolic inflexibility consistent with that observed in obesity and T2D. 

Evidence suggests that the microbiome can be selectively modulated in a little as 5 days and that consumption of the prebiotic inulin may influence gut microbiome composition and function by selectively targeting bacteria beneficial for gut health and barrier function [15,16,17]. One way that prebiotics may improve gut health is by promoting increases in several bacterial taxa, such as *Bifidobacteria* [16,18]. In turn, this might lead to improved gut-barrier function, reduced endotoxin concentration, lower levels of pro-inflammatory cytokines, and reduced risk of T2D with inulin consumption [15,16,19,20,21]. 

Supplementation with the prebiotic inulin may be a simple and efficacious strategy to decrease the risk for T2D. However, there is limited evidence evaluating the mechanisms by which prebiotic supplementation with inulin may alter glucose metabolism and diminish T2D risk in humans [21,22]. Therefore, the objective of this proof-of-concept pilot randomized controlled trial (RCT) was to evaluate this possibility using a rigorous controlled feeding design [23]. We hypothesized that inulin supplementation would improve peripheral insulin sensitivity and in vivo skeletal muscle substrate preference in adults at risk of T2D [23]. We further hypothesized that improvements, if observed, would be associated with increased abundance of *Bifidobacteria* as well as reduced intestinal permeability and circulating endotoxin concentrations.

## 2. Materials and Methods

### 2.1. Participants 

**Figure 1:**
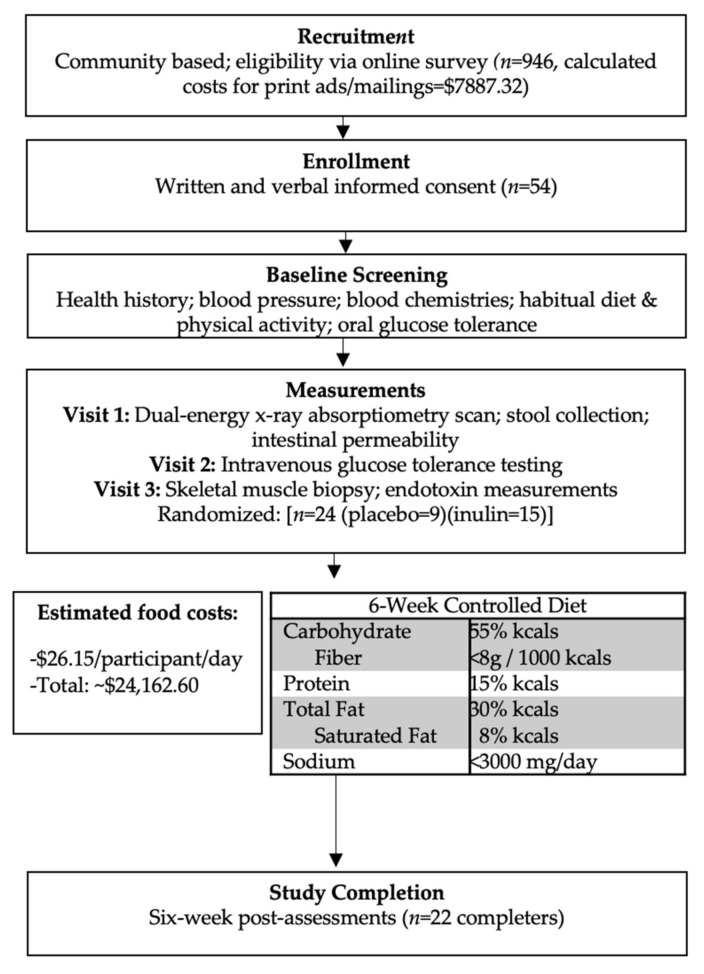
Study Protocol Overview.

**Figure 2:**
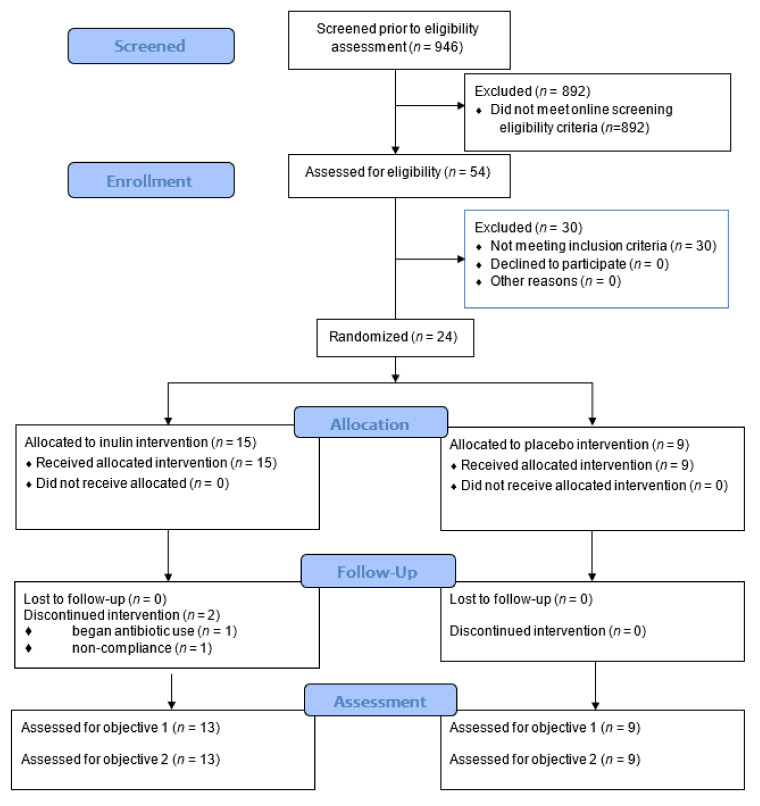
CONSORT Flow Diagram (Pilot Extension) [26].

Detailed rationale and methods for this pilot RCT were previously described [23]. An overview of our study protocol is presented in Figure 1. Briefly, 946 individuals were screened for participation, and 24 individuals were consented, screened, and randomized in this trial (Figure 1 and Figure 2). Participants were between 40–75 years old with a body mass index (BMI) between 25–39.9 kg/m^2^ and sedentary to recreationally active [24]. Body weight was stable for all participants 6 months prior to study enrollment, and none of the participants had taken antibiotics, fiber supplements, multivitamin supplements, or antioxidants within the 3 months preceding enrollment. In addition, all participants met 1 or more of the following criteria established for elevated T2D risk: American Diabetes Association (ADA) risk screener score ≥5; hemoglobin A1c (HbA1c) between 5.7–6.4 mg/dL; fasting blood glucose (FBG) between 100–125 mg/dL; or 2-h oral glucose tolerance test (OGTT) value between 140–200 mg/dL [2,25]. This trial was registered on clinicaltrials.gov (Identifier: NCT02346838) and approved by Virginia Tech. Institutional Review Board (protocol #13-694). All participants provided verbal and written informed consent prior to participation. 

### 2.2. Experimental Design

This trial was a randomized, double-blind placebo-controlled feeding trial of inulin (10 g/day [Frutafit^®^ IQ, Sensus American, Inc., Lawrenceville, NJ, USA; 100% chicory root inulin, DP 9–12]) or placebo (maltodextrin; 10 g/day) supplementation for 6 weeks. The inulin dose selected was based on tolerability and anticipated metabolic benefit [27]. Randomization and enrollment, stratified by sex, was performed using a computerized random number generator by an individual (BMD) not involved in the collection or analysis of the data. Measurements were performed at baseline (visits 1–3; Figure 1) and following the 6-week intervention in the Human Integrative Physiology Laboratory. All participants consumed an isocaloric standardized diet (55% carbohydrate [<8 g fiber/1000 kcals], 30% fat [8% saturated fat], 15% protein) (Figure 1, Table S1) with daily supplementation of either inulin or placebo until follow-up testing was completed. Supplementation vials were labeled A or B to keep study personnel and participants blinded to group assignment. Participants reported to the Metabolic Kitchen and Dining Laboratory a minimum of 3 days/week to collect provided meals and undergo body weight checks. During these visits, participants consumed a supervised breakfast meal, which included consumption of the 10 g supplement (inulin or placebo) dispersed in 16 fluid ounces of water and was then provided with coolers containing their study food for the next 48 h. Coolers from the previous 2 days were collected by research personnel, and any uneaten food was weighed. 

### 2.3. Experimental Testing

All testing sessions during screening, baseline, and week 6 were completed between the hours of 5:00 a.m. and 10:00 a.m. in the fasted state. At baseline testing, participants were provided a list of instructions that included abstaining from alcohol, caffeine, and physical activity (PA) for 48 h prior to the scheduled testing visit. Participants were free from self-reported acute illness for a minimum of 2 weeks prior to all scheduled testing visits.

### 2.4. Measurements and Procedures

Body weight and height were measured on a digital scale (nearest 0.l kg) and mounted stadiometer, respectively (Scale-Tronix Model 5002, White Plains, NY, USA). Body composition was measured utilizing dual-energy x-ray absorptiometry (General Electric, Lunar Digital Prodigy Advance, software version 8.10e Madison, WI, USA). Blood chemistries were performed in a CLIA certified laboratory (Solstas Lab Partners, Roanoke, VA, USA). Blood pressure was measured using an automated oscillometric device (GE Dynamap Carescape V100, GE Healthcare, Chicago, IL, USA). Habitual dietary intake was assessed using self-reported 4-day food intake records. A trained research technician provided study participants with written and verbal instruction for accurately measuring and recording food intake. The Nutrition Data Systems for Research software (NDS-R 2014, University of Minnesota, Minneapolis, MN, USA) was used to estimate energy and macronutrient content from the dietary records. Habitual physical activity (PA) level was measured by an accelerometer (GT1M, Actigraph, Pensacola, FL, USA) worn for 4 consecutive days before and after the intervention.

Peripheral insulin sensitivity, i.e., insulin sensitivity index (SI), glucose effectiveness (Sg), acute insulin response to glucose (AIRg), and disposition index (DI) were measured using a frequently sampled intravenous glucose tolerance test (IVGTT) with Bergman’s minimal model (MINMOD Millennium) [28], as described previously [23,29]. IVGTT findings were presented for 8 participants in the placebo group and 9 in the inulin group due to missing blood samples that precluded performing the minimal model analysis. Briefly, an intravenous catheter was placed, and baseline blood samples were obtained at [(I)= −10 and −1 min. Dextrose (0.3 g/kg; 50% solution) was injected at time 0 and insulin (0.025 U/kg) was injected at (*t*)= 20 min. Subsequent samples were obtained at (*t*) = 1, 2, 3, 4, 5, 6, 7, 8, 10, 12, 14, 16, 18, 22, 23, 24, 25, 27, 30, 40, 50, 60, 70, 80, 90, 100, 120, 150, and 180 min during the 3 h protocol. Plasma glucose samples were analyzed using a YSI Glucose Analyzer 2300 Stat Plus (Yellow Springs, OH, USA), and serum insulin concentration was analyzed via ELISA (ALPCO Diagnostics, Salem, NH, USA). HOMA-IR was calculated from fasting insulin and glucose concentrations according to Levy et al. [30].

Serum endotoxin concentrations were determined using the PyroGeneTM Recombinant Factor C Endotoxin Detection Assay (Lonza International, Basel, Switzerland). Lipopolysaccharide binding protein concentration was measured by ELISA (R&D Systems, Minneapolis, MN, USA). 

Skeletal muscle biopsies were obtained from the vastus lateralis using a modified Bergstrom needle technique with suction [31,32]. Glucose and pyruvate oxidation were evaluated by measuring ^14^C-CO_2_ production. Complete and incomplete fatty acid oxidation were assessed by measuring ^14^C-CO_2_ and acid soluble metabolites. Skeletal muscle substrate preference was determined by [1-^14^C] pyruvate oxidation with and without the availability of non-labeled palmitic acid. The suppressibility of pyruvate oxidation in the presence of palmitic acid compared to control (without palmitic acid) was expressed as the ratio of pyruvate oxidation—free fatty acids: pyruvate oxidation + free fatty acids. All substrate metabolism measures were assessed using skeletal muscle homogenates from biopsied tissue and were previously described [14,23,33,34]. In addition, citrate synthase and cytochrome-c oxidase mitochondrial enzyme activities were measured utilizing the biopsied muscle samples [35,36].

Stool collection kits (Omnigene gut for microbiome, Owatonna, ON, Canada) were provided to participants at each testing timepoint. Bacterial DNA was isolated from fecal samples using the QIAamp^®^ PowerFecal^®^ DNA Kit from QIAGEN (Hilden, Germany). All extractions from 0.25g of feces were completed by manufacturer’s instructions. The resulting elutions were then evaluated for DNA yield using a NanoDrop™ 2000 Spectrophotometer (Waltham, MA, USA) and stored at −80 °C. 

The number of copies of Bifidobacterium 16s rRNA gene in each sample was quantified using quantitative real-time PCR methods using primer sets Bif164-f (5′- GGG TGG TAA TGC CGG ATG -3′) and Bif662-r (5′- CCA CCG TTA CAC CGG GAA -3′) [37,38,39]. Each reaction contained 50 ng/μL sample DNA, 15 uL of qRT-PCR master mix (BioRAD SSOAdvanced™ Universal Inhibitor-Tolerant SYBR^®^ Green Supermix), 2.5 μL of Bif166-f primer, 2.5 μL of Bif662-r primer, and filled to 30 μL volume with nuclease-free water. qRT-PCR conditions were as follows: 1 cycle of amplification at 95 °C for 3 min, 40 cycles at 95 °C for 30 s, 62 °C for 40 s, and 72 °C for 1 min. The melt curve was established by heating at 0.5 °C increments from 62 to 95 °C. qRT-PCR assays were conducted using a BioRad (Hercules, CA, USA) CFX thermal cycler. 

Samples were quantified based on extrapolation to a standard curve generated using g-block^®^ Gene Fragments from Integrated DNA Technologies (Coralville, IA, USA) to serve as a standard curve. The gene fragments used were designed as copies of the genus specific *Bifidobacteria* 16S rDNA gene. G-block stock was diluted to 100 ng/μL using nuclease-free water, then pipetted into microcentrifuge vials in a serial 9-fold dilution from 10^−1^ to 10^−8^. 8.9 × 10^11^ copies of the *Bifidobacteria* 16S rRNA gene were determined to be present in g-block^®^ stock. Blanks containing nuclease-free water only were run as negative controls. 

A defined mix of 4 sugar probes followed by 24 h urine collection to assess intestinal permeability were completed at each testing timepoint, and total urine volume was recorded. Intestinal permeability for the upper and lower gastrointestinal tract was calculated and analyzed as % urinary excretion and excretion ratios of urinary sugars [40,41,42,43,44]. Sugars were measured by UPLC-MS as previously described [23]. Furthermore, intestinal permeability was divided into gastroduodenal (expressed as: 0–5 h % sucrose excretion and sucrose-mannitol ratio), small intestinal (lactulose-mannitol ratio 0–5 h and 6–24 h), and colonic permeability (expressed as: 0–5 h and 6–24 h % sucralose excretion and sucralose-mannitol ratio). Plasma endotoxin concentrations were determined using the PyroGeneTM Recombinant Factor C Endotoxin Detection Assay (Lonza International).

### 2.5. Adverse Events and Side Effect Monitoring

During the controlled diet phase of the investigation, participants were instructed to alert study personnel of any atypical gastrointestinal symptoms or side effects (e.g., gas, bloating, or diarrhea), which were reported on a standardized questionnaire [27,45]. 

### 2.6. Calculations and Statistical Analyses

Compliance to the controlled diet was calculated for each food provided to each participant for all 6 weeks (calculated as: [provided food weight—consumed weight]/provided weight × 100 = %compliance).

Independent *t*-tests were used to test for differences in baseline participant characteristics. Two-way repeated-measures analyses of variance were used to test for main effects for group, time, and the group × time interaction. Analyses of Covariance (ANCOVA) were utilized to adjust for baseline, where baseline and week-6 were input into the ANCOVA model as the independent and dependent variables, respectively and inulin and placebo were included as independent classification variables. Pearson’s product-moment correlations were used to assess relationships among variables. SPSS Statistical Software (version 26, 2019; IBM, Armonk, NY, USA) and SAS Enterprise Guide (version 7.1; IBM, Cary, NC, USA) was used for all analyses.

### 2.7. Sample Size

The purpose of this pilot trial was to establish proof-of-concept efficacy and to obtain preliminary data for a larger trial. We calculated the sample size needed to detect a physiologically and statistically significant improvement in insulin sensitivity with inulin supplementation using G*Power 3.1 [46]. With 2 groups, 2 repeated measures, and alpha = 0.05, we estimated that we would have 90% power to detect a 20% increase in insulin sensitivity (effect size = 0.61) with *n* = 24 participants per group. However, the trial was terminated at the end of the funding period when an interim analysis was performed, indicating that the effect size for the change in peripheral insulin sensitivity was much smaller in magnitude (0.21) than originally estimated. The results of that analysis are presented herein.

## 3. Results

Of the 54 individuals who were consented and screened in the laboratory, 24 individuals met all inclusion criteria and were randomized (Figure 1 and Figure 2). Recruitment costs were estimated to be ~$8000 (~$333 per participant randomized; Figure 1). A total of 22 individuals completed the trial (92% retention).

### 3.1. Baseline Participant Characteristics

Baseline characteristics are summarized in Table 1. There were no group differences in participant characteristics at baseline (all *p* > 0.05) except fasting insulin concentration was higher (*p =* 0.027) in the inulin compared with the placebo group. Participants were primarily Caucasian females (65%). All participants scored a 5 or higher on the ADA risk screener and were considered obese based upon BMI classification and body fat percentage. 

### 3.2. Controlled Diet

The composition of the controlled diet is shown in Figure 1 and Table S1. Daily food costs were estimated to be $26.15 per participant (Figure 1). Overall compliance to the controlled diet was 97.5% (inulin, 98% compliance; placebo, 97% compliance). In addition, there were no changes in body weight or PA with the intervention (Figure S1 and Table S2; all *p* > 0.05). 

### 3.3. Side Effects

One participant in each group (2 total) reported mild gastrointestinal side effects that included bloating and loose stool. However, these side effects resolved within 48 h of initial dosing. 

### 3.4. Insulin Sensitivity, Skeletal Muscle Substrate Oxidation, and Mitochondrial Enzyme Activities 

**Figure 3:**
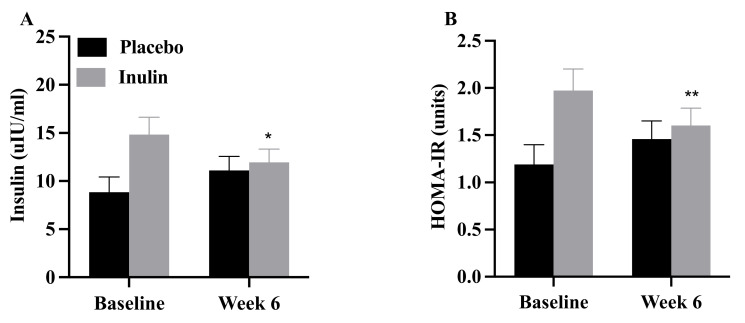
Values are mean ± SEM. There was a significant reduction in (**A**) fasting insulin and (**B**) HOMA-IR in the inulin group. HOMA-IR = homeostatic model of assessment for insulin resistance. * *p* = 0.03; ** *p* = 0.004.

FBG did not change (*p* > 0.05) with the intervention in the placebo (baseline: 87 ± 10 mg/dL, 6 weeks: 85 ± 8 mg/dL) or inulin (baseline: 94 ± 10, 6 weeks: 97 ± 13 mg/dL) group. However, fasting insulin concentrations and HOMA-IR declined in the inulin but not the placebo group (Figure 3). The magnitude of reduction in fasting insulin concentration was attenuated following adjustment for baseline concentrations; wherein, baseline was utilized as the independent variable (*p =* 0.04). The reduction in HOMA-IR was no longer significant after adjustment for baseline levels. There were no changes in SI or any of the other IVGTT-related variables following the intervention (Figure S2; all *p* > 0.05). In addition, there were no changes in glucose oxidation, fat oxidation, pyruvate oxidation, or substrate preference in homogenates with the intervention (Table 2; all *p* > 0.05). Skeletal muscle mitochondrial enzyme activities also remained unchanged (*p* > 0.05).

### 3.5. Bifidobacteria, Intestinal Permeability, and Endotoxin Concentrations 

**Figure 4:**
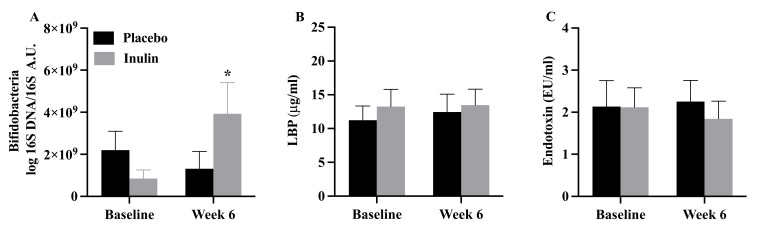
Values are mean ± SEM. (**A**) *Bifidobacteria* increased following 6-weeks in the inulin, but not the placebo (*p* = 0.04) following the intervention. There was no significant change in (**B**) lipopolysaccharide binding protein or (**C**) endotoxin concentration in the placebo or inulin groups (both *p* > 0.05). LBP = lipopolysaccharide binding protein. * *p* = 0.04.

There were no group differences in *Bifidobacteria* copy number, intestinal permeability, endotoxin or lipopolysaccharide binding protein concentrations at baseline (all *p* > 0.05). *Bifidobacteria* increased with inulin supplementation but not in the placebo group (Figure 4A (*p =* 0.04)). There were no changes in intestinal permeability (Table 3), plasma endotoxin concentration, or lipopolysaccharide binding protein concentration (Figure 4B,C (all *p* > 0.05)). There was no significant correlation between changes in *Bifidobacteria* and any of the other outcome variables.

## 4. Discussion

The new finding of the present pilot study is that supplementation with inulin did not alter peripheral insulin sensitivity or skeletal muscle metabolic flexibility despite increases in gut *Bifidobacteria*. Intestinal permeability and endotoxin concentrations did not change following the intervention. The reduction in fasting insulin concentrations and HOMA-IR with inulin supplementation appears to be associated with higher baseline levels.

The improvement in HOMA-IR, an index of hepatic insulin resistance [47] following inulin supplementation is consistent with some [48,49] but not all prior studies [21,22,50,51]. The mechanism(s) responsible is(are) unclear; short-chain fatty acids produced via gut bacteria fermentation of dietary fibers have been implicated [47,50]. However, the greater reduction in HOMA-IR in the inulin group is difficult to interpret and may have been confounded by the corresponding higher baseline levels in this group compared with placebo. Taken together, our findings suggest that inulin supplementation had no obvious impact on peripheral insulin sensitivity, but the impact on HOMA-IR in adults with overweight and obesity at increased risk of T2D in the present study is unclear.

Compared to previous studies, our data support that fasting insulin concentration declined following inulin supplementation [21,49], however, a multitude of studies did not observe this trend [22,48,50,51,52,53]. Although the metabolic impacts of inulin consumption have not been fully elucidated, a reduction in insulin secretion, an increase in insulin clearance, or both may have contributed. The higher baseline insulin concentration, as described for HOMA-IR, is similarly difficult to interpret. Nevertheless, AIRg did not change following inulin supplementation in the present study, suggesting that perhaps enhanced insulin clearance was responsible for the reduction in fasting insulin concentrations. Future studies will be necessary to test this hypothesis and should consider the addition of satiety hormone measurements and c-peptide assays to further differentiate changes in insulin secretion versus clearance.

The inulin dosage selected for this study was based upon the reproducibility of its bifidogenic effect and tolerability (e.g., minimization of gastrointestinal distress, which may induce gut dysbiosis) [16,54,55]. The doses utilized in previous studies ranged from 10 g/d to 30 g/d, but there was no clear pattern of a dose-response effect. Our participants were categorized as overweight/obese and were considered at high risk for T2D based upon the ADA risk screener [2]. In addition, body weight was kept stable during the controlled feeding intervention to avoid the potential impact of weight loss on insulin sensitivity. Findings on the direction of change for weight loss and glucose homeostasis with inulin supplementation were mixed with some studies reporting concurrent improvements in body weight and insulin sensitivity [21,49], while others report no change in body weight [48] or did not report body weight at all [55,56,57]. Although we observed that HOMA-IR declined in the absence of weight loss, these changes were diminished after correcting for baseline values. While there may be a small impact of inulin supplementation, we cannot exclude the possibility that the reduction in HOMA-IR was due to regression to the mean.

There were no changes in intestinal permeability, endotoxin concentrations (lipopolysaccharide binding protein), or in vivo substrate oxidation following inulin supplementation in the present study despite a significant bifidogenic effect. The lack of impact of inulin supplementation on intestinal permeability and endotoxin concentrations is inconsistent with prior studies [49,58]. The reasons for this are not clear. Dehghan et al. [49] reported a reduction in endotoxin concentration following inulin supplementation in women with T2D, suggesting intestinal permeability may have been reduced. As such, one possibility is that because our participants were relatively healthy (i.e., did not have impaired glucose tolerance or T2D) there was less to intervene upon. However, Russo et al. [59] reported that supplementation with inulin-enriched pasta reduced intestinal permeability and zonulin concentrations and increased glucagon-like peptide 2 in healthy adults; there was no change in these variables following supplementation with the control pasta. Future studies will be necessary to clarify this issue. 

The strengths of our investigation include the rigorous RCT design with controlled feeding, high participant retention rate and compliance to the controlled diet. As intended, the participant PA levels and both body weight and composition did not change over the course of the study. In addition, the inulin supplementation at a dosage of 10 g/d produced a significant increase in Bifidobacteria and was well-tolerated by the participants. However, we acknowledge the limitations of this trial as well, including that the sample size was small due to substantial recruitment costs, racially homogenous, and most participants were female. Furthermore, our ability to fully sequence the gut microbiome was compromised by budgetary considerations. Future studies will require larger and more heterogenous samples. In addition, most participants were included based upon scores from the ADA risk screener rather than prediabetes clinical criteria. Although they were classified as overweight or obese and at increased risk for T2D, our participants were relatively healthy; this may have impacted the outcome of the study. 

### Implications for the Future

Our interim analysis indicated that the effect size for the change in peripheral insulin sensitivity was much smaller in magnitude (0.21) than originally estimated. With this effect size, 564 participants would be required to detect group differences in our primary outcome. Thus, our trial was terminated at the end of the funding period. Although not our primary or secondary aim, given the pilot nature of this research, it is worthwhile to address several aspects of trial design feasibility, including recruitment, retention, controlled diet delivery, adherence to outpatient controlled feeding, and tolerability of inulin dosage [26]. Participant retention was high at 92%, despite the high participant burden associated with controlled feeding, such as multiple weekly visits to the metabolic kitchen for body weight assessment and food pickup. Costs for delivering the controlled diets were consistent with that budgeted. Dietary adherence according to food return weigh-backs was high with ~98% of foods provided consumed, and weight stability was maintained as intended. The tolerability of the inulin dosage used was supported with only one inulin group participant reporting mild GI side effects. However, recruitment feasibility was challenged by the screening criteria and higher-than-anticipated costs for participant enrollment ($333 per participant randomized), which suggests the need for substantial recruitment budgeting for this type of trial. Given the rigorous controlled feeding design, high dietary compliance, and body weight stability, these findings and in particular, the effect size, suggest that larger-scale studies in a similar study population may not be justified despite the demonstrated feasibility of most aspects of the study design.

## 5. Conclusions

Simple and effective dietary strategies are needed to reduce the incidence and prevalence of type 2 diabetes in the U.S. The findings from this pilot RCT suggest that inulin supplementation does not improve peripheral insulin sensitivity in adults at risk for T2D, at least when body weight and composition are stabilized with controlled feeding. Taken together with the relatively limited available evidence in adults at risk for T2D, the findings of our pilot RCT suggest that in the absence of weight loss there was no clear impact of inulin supplementation on glucose homeostasis. These findings call into question the need for larger-scale investigations in this study population. 

## Figures and Tables

**Table 1:** Baseline participant characteristics.

Descriptives	Placebo (*n* = 9)	Inulin (*n* = 13)
Sex	Males = 3 Female = 6	Males = 5 Female = 8
Race	Caucasian = 9	Caucasian = 12 African = 1
ADA Risk Score	5 ± 0	5 ± 0
Age (years)	54.2 ± 3.2	54.5 ± 2.1
Anthropometrics		
Height (cm)	168.7 ± 3.0	169.2 ± 3.0
Weight (kg)	89.3 ± 3.0	89.5 ± 3.9
BMI (kg/m^2^)	31.2 ± 0.8	31.4 ± 0.9
Body fat (%)	42.3 ± 9.9	40.1 ± 6.7
Blood Chemistries and Blood Pressure		
FBG (mg/dL)	90 ± 4	96 ± 4
Fasting Insulin uIU/mL	9 ± 2	15 ± 2 *
2-hr glucose (mg/dL)	118 ± 17	121 ± 12
HbA1c (%)	5.7 ± 0.1	5.4 ± 0.1
TC (mg/dL)	209 ± 10	215 ± 8
HDL (mg/dL)	57 ± 6	50 ± 3
LDL (mg/dL)	123 ± 14	138 ± 9
VLDL (mg/dL)	29 ± 7	27 ± 3
TG (mg/dL)	147 ± 33	134 ± 14
SBP (mmHg)	128 ± 3	130 ± 3
DBP (mmHg)	79 ± 3	77 ± 2
Habitual Dietary Intake		
Kcals	2119 ± 191	2094 ± 165
Protein (grams) (% energy)	79 ± 8 15 ± 0	98 ± 6 19 ± 0
Carbohydrates (grams) (% energy)	258 ± 24 49 ± 1	227 ± 18 43 ± 0
Fats (grams) (% energy)	85 ± 11 36 ± 1	92 ± 9 40 ± 0
Dietary fiber (g)	22 ± 2	17 ± 2
Soluble fiber (g)	7 ± 1	7 ± 1
Pectins (g)	2 ± 0	2 ± 0
Sodium (mg)	3166 ± 279	3699 ± 270

Data are mean ± SEM descriptive statistics. Abbreviations used: ADA = American Diabetes Association; BMI = body mass index; FBG = fasting blood glucose; HbA1c = hemoglobin A1c; TC = total cholesterol; HDL = high-density lipoproteins; LDL = low-density lipoproteins; VLDL = very low-density lipoproteins; TG = triglycerides; SBP *=* systolic blood pressure; DBP *=* diastolic blood pressure; kcals = kilocalories. * *p* = 0.027.

**Table 2:** Fasted participant skeletal muscle metabolism and mitochondrial enzyme variables before and after 6-weeks of supplementation with placebo or inulin.

Variable	Placebo		Inulin		Interactions
	Baseline	Week 6	Baseline	Week 6	*p*-Values
Glucose Oxidation	5.8 ± 1.0	5.5 ± 1.0	5.3 ± 1.0	5.9 ± 1.1	*p =* 0.90
Fatty Acid Oxidation	6.9 ± 1.1	7.4 ± 0.7	7.0 ± 1.0	7.6 ± 1.0	*p =* 0.22
Pyruvate Oxidation	354.5 ± 36.5	339.9 ± 45.9	248.9 ± 23.4	285 ± 29.6	*p =* 0.43
Metabolic Flexibility	32.4 ± 3.8	23.1 ± 4.0	22.5 ± 4.4	31.5 ± 3.8	*p =* 0.07
Citrate synthase	52.5 ± 8.2	53.3 ± 10.0	41.6 ± 7.6	39.8 ± 5.0	*p =* 0.52
Cytochrome-c Oxidase	139.1 ± 25.0	171.8 ± 40.2	102.7 ± 20.7	132.8 ± 15.9	*p =* 0.50

All values are expressed as mean ± SEM. All interactions, derived from 2-way repeated-measures ANOVA, were non-significant (*p* > 0.05). Units for skeletal muscle metabolism are expressed as: (μmol/mg protein/hour). Metabolic flexibility is expressed as the ratio of pyruvate oxidation ± free fatty acids.

**Table 3:** Intestinal permeability outcomes and change over time.

Variable	Placebo			Inulin			Interactions
	Baseline	Week 6	Δ-Score	Baseline	Week 6	Δ-Score	*p*-Values
**Small intestine permeability**							
**0–5 h (ratio)**	0.0117 ± 0.001	0.0096 ± 0.0013	−0.0021 ± 0.0018	0.0109 ± 0.0014	0.0090 ± 0.0010	−0.0018 ± 0.0011	*p* = 0.88
**6–24 h (ratio)**	0.0342 ± 0.0065	0.0266 ± 0.0043	−0.0076 ± 0.0058	0.0323 ± 0.0053	0.0218 ± 0.0032	−0.0105 ± 0.0040	*p* = 0.68
**Gastroduodenal permeability**							
**0–5 h (%)**	0.0241 ± 0.0060	0.0289 ± 0.0160	0.0048 ± 0.0170	0.0226 ± 0.0041	0.0326 ± 0.0114	0.0010 ± 0.0010	*p* = 0.78
**0–5 h (ratio)**	0.0014 ± 0.0004	0.0017 ± 0.0010	0.0004 ± 0.0010	0.0013 ± 0.0002	0.0013 ± 0.0010	0.0001 ± 0.0010	*p* = 0.72
**Colonic permeability**							
**0–5 h (%)**	1.3910 ± 0.2642	1.2620 ± 0.3510	−0.1292 ± 0.5634	1.4440 ± 0.4707	1.0520 ± 0.2723	−0.3920 ± 0.4739	*p* = 0.72
**0–5 h (ratio)**	0.0863 ± 0.0208	0.0833 ± 0.0238	−0.0030 ± 0.0333	0.0817 ± 0.0706	0.0490 ± 0.0083	−0.0327 ± 0.0197	*p* = 0.43
**6–24 h (%)**	6.4160 ± 0.8898	6.3550 ± 2.718	−0.0608 ± 2.8890	5.4930 ± 1.0450	3.1860 ± 1.2270	−2.3070 ± 0.9152	*p* = 0.42
**6–24 h (ratio)**	0.5011 ± 0.0905	0.4764 ± 0.1703	−0.0248 ± 0.2078	0.3616 ± 0.0694	0.2121 ± 0.0910	−0.1496 ± 0.0826	*p* = 0.55

Values are mean ± SEM. All interactions, derived from 2-way repeated measures ANOVAs, were non-significant (*p* > 0.05) for small intestinal, gastroduodenal, and colonic permeability in the placebo and inulin groups. Δ calculation = (week 6 excretion ratio—baseline excretion ratio = Δ-score) % excretion: (total excretion/provided dose) ∗ 100 Permeability ratios: Small intestinal—lactulose:mannitol; Gastroduodenal—sucrose:mannitol; Colonic—sucralose:mannitol.

## Data Availability

Data and materials are made available by request and at the discretion of the corresponding author.

## Supplementary Materials

The following are available online at https://www.mdpi.com/article/10.3390/nu13093235/s1, Table S1. Controlled Diet Composition; Table S2. Habitual Physical Activity; Figure S1. Body Weight; Figure S2. IVGTT-Related Variables. (NOTE: for review, included in this file following the references).
