# Supplementary material for: Prebiotic Inulin Supplementation and Peripheral Insulin Sensitivity in adults at Elevated Risk for Type 2 Diabetes: A Pilot Randomized Controlled Trial
Source: Nutrients. 2021 Sep 17;13(9):3235. doi: 10.3390/nu13093235 (PMC8471706; doi:10.3390/nu13093235)
Supplement: Supplementary file 1 [file nutrients-13-03235-s001.zip › nutrients-1326331-supplementary.pdf]

Supplemental Tables and Figures

Table S1. Controlled Diet Composition.

Variable	Standardized Composition
**Carbohydrate**	55% kcals
**Protein**	15% kcals
**Total Fat**	30% kcals
**Saturated Fat**	8% kcals
**Fiber**	<8g / 1000 kcals
**Sodium**	<3,000 mg/day

All variables were standardized across calorie levels.

Table S2. Habitual physical activity.

Variable	Placebo (n=9)		Inulin (n=13)	
	Baseline	Week 6	Baseline	Week 6
**Sedentary time (min/d)**	534 ± 42	422 ± 31	586 ± 39	563 ± 33
**Light PA (min/d)**	304 ± 34	205 ± 25	242 ± 38	248 ± 19
**Moderate PA (min/d)**	36 ± 10	3 ± 11	28 ± 8	21 ± 5
**Vigorous PA (min/d)**	3 ± 3	0 ± 1	0 ± 0	1 ± 1

Data are mean ± SEM. There were no changes in any of the PA variables with intervention in the placebo and inulin groups (all p>0.05). Abbreviations used: PA = physical activity.

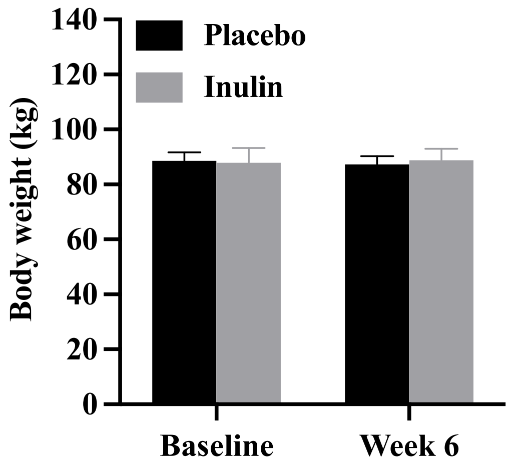

Figure S1. Data are mean±SEM. There were no differences in body weight with the intervention in the placebo or inulin groups (all p>0.05)

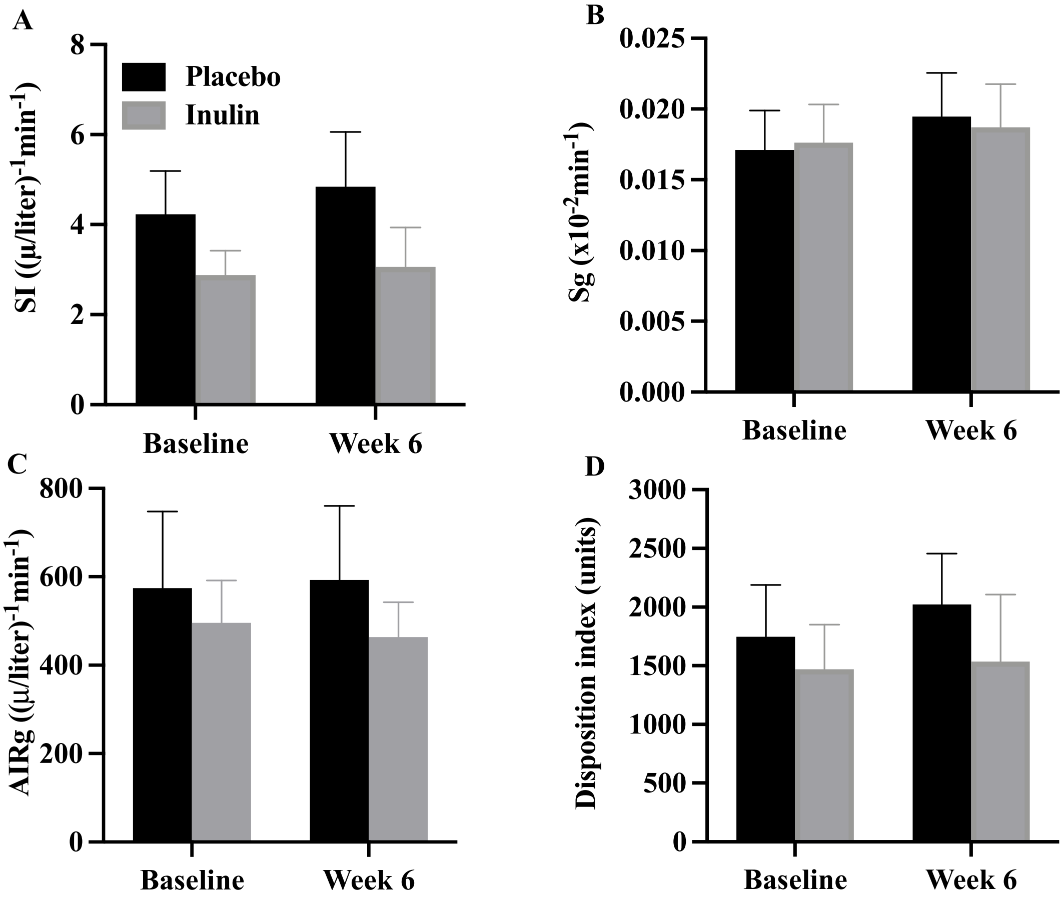

Figure S2. Data are mean+/- SEM. There were no changes in any of the IVGTT-related variables with the intervention in the placebo or inulin groups (all p>0.05). A, SI=insulin sensitivity; B, Sg=glucose effectiveness; C, AIRg=acute insulin response to glucose; D, Disposition index.
